# Effects of controlled ovarian stimulation on vascular barrier and endothelial glycocalyx: a pilot study

**DOI:** 10.1007/s10815-021-02233-x

**Published:** 2021-07-20

**Authors:** Nikolai Hulde, N. Rogenhofer, F. Brettner, N. C. Eckert, I. Fetz, J-I. Buchheim, T. Kammerer, A. Dendorfer, A. Choukèr, K. F. Hofmann-Kiefer, M. Rehm, C. Thaler

**Affiliations:** 1 Department of Anesthesiology, Heart and Diabetes Center North Rhine-Westphalia, Ruhr University Bochum, Georgstr 11, 32545 Bad Oeynhausen, Germany; 2 Division of Gynecological Endocrinology and Reproductive Medicine, Department of Gynecology and Obstetrics, Ludwig-Maximilians University, Marchioninistr. 15, 81377 Munich, Germany; 3 Department of Anesthesiology, Ludwig-Maximilians University, Marchioninistr. 15, 81377 Munich, Germany; 4 Walter-Brendel-Centre of Experimental Medicine, Hospital of the University Munich, Ludwig-Maximilians University, Marchioninistr. 15, 81377 Munich, Germany

**Keywords:** Controlled ovarian stimulation;, Assisted reproduction, Ovarian hyperstimulation syndrome, Endothelial glycocalyx, Ovulatory cycle, Sexual hormones

## Abstract

**Purpose:**

Controlled ovarian stimulation significantly amplifies the number of maturing and ovulated follicles as well as ovarian steroid production. The ovarian hyperstimulation syndrome (OHSS) increases capillary permeability and fluid extravasation. Vascular integrity intensely is regulated by an endothelial glycocalyx (EGX) and we have shown that ovulatory cycles are associated with shedding of EGX components. This study investigates if controlled ovarian stimulation impacts on the integrity of the endothelial glycocalyx as this might explain key pathomechanisms of the OHSS.

**Methods:**

Serum levels of endothelial glycocalyx components of infertility patients (n=18) undergoing controlled ovarian stimulation were compared to a control group of healthy women with regular ovulatory cycles (n=17).

**Results:**

Patients during luteal phases of controlled ovarian stimulation cycles as compared to normal ovulatory cycles showed significantly increased Syndecan-1 serum concentrations (12.6 ng/ml 6.11^25th^–19.13^75th^ to 13.9 ng/ml 9.6^25th^–28.9^75th^; p=0.026), indicating shedding and degradation of the EGX.

**Conclusion:**

A shedding of EGX components during ovarian stimulation has not yet been described. Our study suggests that ovarian stimulation may affect the integrity of the endothelial surface layer and increasing vascular permeability. This could explain key features of the OHSS and provide new ways of prevention of this serious condition of assisted reproduction.

## Introduction

Controlled ovarian stimulation (COS) is an important element of many assisted reproductive treatment modalities as it provides increased metaphase II oocyte numbers and significantly improves pregnancy rates. It is generally accompanied by amplified ovarian steroidogenesis. Both estradiol and progesterone concentrations during COS significantly exceed the levels measured during monofollicular cycles [1, 2]. A rare, but serious complication of COS is ovarian hyperstimulation syndrome (OHSS) that is characterized by increased capillary permeability, fluid extravasation, augmented coagulation, and hemoconcentration. OHSS may become life-threatening secondary to thromboembolism or compromised pulmonary or cardiovascular function [1–5].

Risk factors for the development of OHSS include high antral follicle count, polycystic ovarian syndrome, high doses of gonadotrophins, high numbers of eggs retrieved, high concentrations of estradiol, and high or repeated doses of hCG as well as early pregnancy after in vitro fertilization [1, 3–5].

Much work has focused on the mechanism leading to OHSS and recent work points to vascular endothelial growth factor (VEGF), a vasoactive glycoprotein that is secreted by granulosa cells and stimulates endothelial cell proliferation and permeability. VEGF levels have been shown to increase in response to FSH and hCG administration and higher VEGF levels were found in patients with higher numbers of eggs retrieved [6].

An important component, which is highly involved in the regulation of the vascular permeability, is the endothelial glycocalyx (EGX). This structure coats endothelial cells of a healthy vascular bed on its luminal side [7–10]. The EGX consists of Syndecan-1, a transmembranous proteoglycan, heparan sulfate and hyaluronic acid, and two cross-linked glycosaminoglycans [10, 11]. Interaction of EGX with albumin and soluble plasma proteins constitutes the active form, the endothelial surface layer (ESL). The very large dimension of the ESL of about 350m^2^ offers a large surface for a multitude of pathophysiological processes [8–12].

Recently, we have shown that ovulatory cycles influence release of EGX components and highest concentrations of Syndecan-1 were demonstrated in luteal phases, suggesting products of the corpus luteum are involved in destabilizing the EGX and increasing vascular permeability [13]. Its degradation is accompanied by shedding of one or more EGX components into the blood. However, the mechanism behind EGX degradation/shedding is still incompletely understood. An activation of the immune system by pro-inflammatory cytokines such as TNF-α leads to a shedding of the EGX and a loss of vascular integrity [14–16]. It is known that free radicals, together with the subsequent activation of matrix metalloproteinases and cell surface endoglycosidases, can also detach various components of the endothelial glycocalyx individually [15, 17–21]. Shedded components of the EGX in turn act as chemotactical stimuli and trigger an activation of polymorphonuclear leukocytes (PMNLs) [14, 16]. It appears intriguing that many risk factors of OHSS involve high numbers and strong intensity of stimulation of corpora lutea.

These considerations led us to ask whether controlled ovarian stimulation influences the release of components of the endothelial glycocalyx and whether there is evidence for the activation of a TNF and PMNL pathway.

## Materials and methods

The current study was authorized by the Review Board of the Ludwig-Maximilians-University (IRB project number: 137-13). Subjects were in a healthy physical and mental condition. Diabetes type I or II, obesity (BMI > 25 kg/m^2^), arterial hypertension, thrombosis, thrombophilia, acute or chronic infections, or vascular diseases led to exclusion, as well as treatment with any kind of medical therapy, performing competitive sports or doing shift work. These parameters were excluded due to potential interaction with EGX components and resulting vascular permeability or controlled ovarian stimulation and OHSS risk [22].

### Control group

The control group consisted of healthy women (n = 21) ovulatory menstrual cycles of 26 to 35 days without hormonal medication during the previous 12 months. Ovulation was determined by positive urinary luteinizing hormone (LH)-self-tests (Clearblue, Wick, Swiss Precision Diagnostics GmbH, Geneva, Switzerland) and was confirmed by measuring serum progesterone (> 10 ng/mL) 8 days after the positive LH-test.

In the control group, blood samples were drawn for baseline measurements on menstrual cycle day 3 (early follicular phase), on the first day with a positive urinary LH-self-test (peri-ovulatory phase) and 8 days thereafter (mid-luteal phase).

### Controlled ovarian stimulation group

This group consisted of infertile women (n = 23) who received controlled ovarian stimulation (COS) with the long gonadotropin-releasing hormone agonist (GnRH-a) protocol undergoing IVF (in vitro fertilization) or ICSI (intracytoplasmatic sperm injection) treatment [23]. Patients were healthy women with BMI≤ 25 kg/m^2^. No physical and mental condition. Diabetes type I or II, obesity (BMI > 25 kg/m^2^). Diabetes, arterial hypertension, thrombosis, thrombophilia, acute or chronic infections, or vascular diseases were excluded. All subjects had their first or second IVF or ICSI without OHSS in previous stimulation protocols. Luteal phase support was done with 3 times 200 mg of micronized progesterone vaginally and started at the day of oocyte retrieval. Serum samples of the COS group were taken on menstrual cycle day 3 (T1) after pituitary downregulation, on day 8 of ovarian stimulation (T2), the day of oocyte retrieval (T3), and seven days after embryo transfer (T4).

### Sample processing

Blood samples were drawn in the morning. After a clotting time of 30 min, the samples were centrifuged (10 min; 1932*g*) and stored at −80 °C. Syndecan-1, heparan sulfate, and hyaluronic acid levels were analyzed with ELISAs according to the manufacturer’s protocols (Diaclone SAS, Besançon, France for Syndecan-1; Echelon Biosciences Inc., Salt Lake City, USA for hyaluronic acid; Fa. Cusabio Art.Nr.: CSB-E09585h for heparan sulfate) [24].

The Institute of Laboratory Medicine tested estradiol, progesterone, and LH-serum levels using electrochemiluminescence. Albumin serum levels were tested using photometric determination and the hemogram was analyzed by flow cytometry.

Fluorescence-activated cell sorting was used to describe the behavior of PMNL as one parameter of non-specific immune response. The spontaneous and inducible hydrogen peroxide release of PMNL was determined by examining their capacity to mount an oxidative burst (hydrogen peroxide release) either in response to receptor-dependent activation by TNF and formyl-methionyl-leucyl-phenylalanine (fMLP) or in response to receptor-independent protein kinase C (PKC) activation though phorbol-12-myristat-13acetate (PMA). The superoxide burst was measured using reactive oxide-mediated reduction of the cell permeant probe, dihydrorhodamine 123 (DHR-123, Molecular Probes), into the fluorescent derivate rhodamine [25, 26].

## Statistics

Mean, standard deviation of the mean, median, and 25th–75th percentile were calculated for each target parameter. To test normality, the Kolmogorov-Smirnov-Test was applied. To compare repeated samples, a repeated measurement analysis of variance (ANOVA), including the Mauchly test and a Greenhouse-Geisser correction, was performed, and followed by a paired t-test. For non-normally distributed data, we used a repeated measurement ANOVA on ranks (Friedman Test). To compare intergroup differences, Kruskal-Wallis and Mann-Whitney U tests were performed for all determinations. A type I error protection of p < 0.05 was considered significant. For this study, Kendall’s tau correlation coefficient was performed for correlation analysis. Statistical analysis was realized in cooperation with the IBE (institution for medical information processing, biometry, and epidemiology of the LMU) and performed using SPSS Version 21, Premium (IBM Corporation, USA).

## Results

**Fig. 1:**
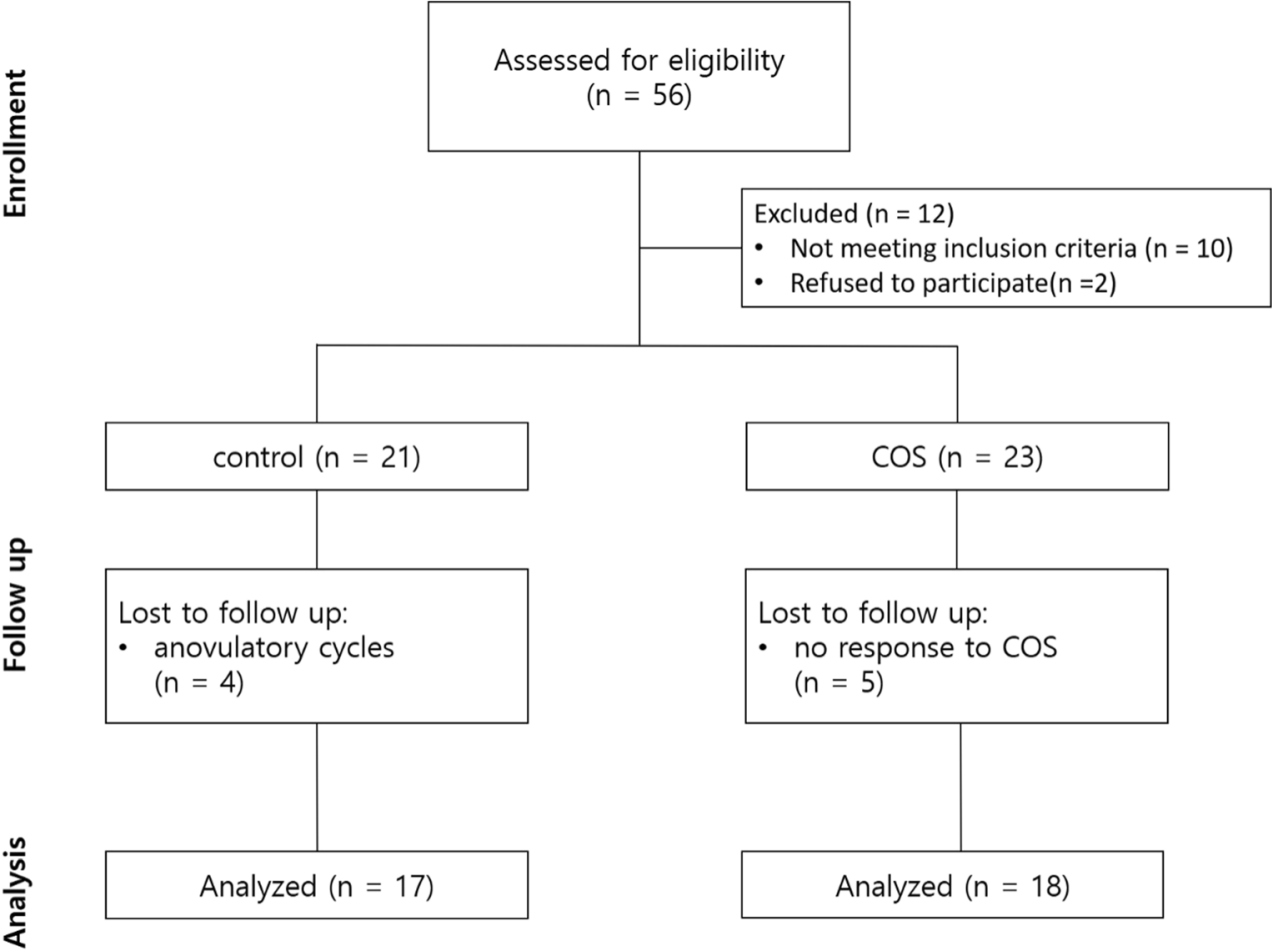
Patient flow diagram. COS: controlled ovarian stimulation group

Thirty-five of the 44 women who were initially enrolled in the study completed the study protocol and were included in the subsequent analysis. The patient flow diagram following the CONSORT criteria is illustrated in Fig. 1. The study population was homogenous regarding height and body weight. In terms of age, the COS group had a significantly higher age than the control group, with a mean age difference of 7.4 years (Table 1).

**Table 1:** Demographic data

Groups (n)	Control (17)	COS (18)	p
Age (years)	29.3 (± 5.4)	36.7 (± 4.2)	0.001
Height (cm)	1.70 (± 0.05)	1.69 (± 0.06)	0.525
Body weight (kg)	65.2 (± 9.1)	71.3 (± 15.1)	0.184
Body mass index (kg/m^2^)	22.5 (± 3.1)	25.0 (± 5.3)	0.273

Data of the subjects enrolled are depicted as mean ± standard deviation

### Estrogen, progesterone and luteinizing hormone

Hormone serum concentrations are summarized in Table 2. The CG group shows ovulatory dynamics of hormones with increased periovulatory LH and estradiol as well as increased luteal progesterone concentrations. The COS group shows significantly increased estradiol from T1 to T3 and high progesterone concentrations at T4. Estrogen and progesterone serum concentrations showed 5–6-times higher levels in the COS group compared to the CG group.

**Table 2:** Hormone serum concentrations

	Estrogen (pg/ml)		Progesterone (ng/ml)		LH (ng/ml)	
	Median	25th–75th	Median	25th–75th	Median	25th–75th
Controlled ovarian stimulation						
T1	15.7	11.8–30.0	0.45	0.3–0.7	3.5	2.1–4.3
T2	581.5	262.8–1016.5	0.6	0.3–0.7	1.3	0.8–2.0
T3	1193.5	842.5–1902.0	7.3	5.8–10.5	0.1	0.1–0.1
T4	1507.5	600.5–2036.8	77.3	33.3–139.3	0.1	0.1–0.1
Control						
Follicular	45.	34.6–55.1	0.4	0.3–0.7	6.3	5.2–9.1
Ovulation	115.0	80.7–348.5	1.1	0.7–2.2	21.4	9.9–41.1
Luteal	235.5	144.5–254.8	12.8	8.7–17.5	6.2	4.0–10.4

*LH* luteinizing hormone

### EGX components

Changes in serum concentrations of the EGX components are shown in Table 3. During the estrogen-dominated phase, serum levels of EGX components remained nearly constant in the CG. Syndecan-1 and heparan sulfate showed decreasing serum levels from T1 to T3, representing the estrogen-dominated phase of the COS therapy. In this phase, hyaluronic acid showed an increase from T1 to T2 followed by a decrease compared to initial values.

**Table 3:** EGX serum concentrations

	Syndecan (ng/ml)			Heparan sulfate (ng/ml)			Hyaluronic acid (ng/ml)		
	Median	25th–75th	p	Median	25th–75th	p	Median	25th–75th	p
Controlled ovarian stimulation									
T1	12.30	9.11–19.05		541.24	464.07–740.575		145.40	99.52–160.41	
T2	13.94	6.20–17.72	0.476	533.52	461.82–578.01	0.170	155.25	131.46–186.33	0.002*
T3	10.96	6.17–15.35	0.035*	491.77	389.99–581.14	0.375	138.73	117.55–166.79	0.031*
T4	13.92	9.56–28.89	0.003*	511.27	441.27–653.36	0.557	143.28	123.10–164.92	0.679*
Control									
Follicular	11.27	4.54–19.49		659.17	507.89–817.94		144.86	119.235–149.23	
Ovulation	10.69	4.01–20.70	0.501	620.84	491.89–730.57	0.177	153.73	123.29–190.06	0.332
Luteal	12.60	6.11–19.13	0.07	751.24	573.76–937.89	0.020*	146.92	138.77–163.572	0.679

Syndecan-1 showed a significant increase from T3 to T4, representing the progesterone-dominated phase of the COS therapy. A medium strong correlation was found between the increase in progesterone and the increase in syndecan-1 (r = 0.294, p = 0.044)/heparan sulfate levels (r = 0.333, p = 0.027). The corresponding phase of the CG also showed a significant increase of Syndecan-1. A comparison of Syndecan-1 serum levels of the CG at luteal phase with serum levels of the COS group at T4 showed significantly higher Syndecan-1 serum levels in the COS group (p = 0.026). The intergroup comparison for heparan sulfate (p = 0.053) and hyaluronic acid (p = 0.756) showed not to be significant.

### Markers of hemoconcentration

**Fig. 2:**
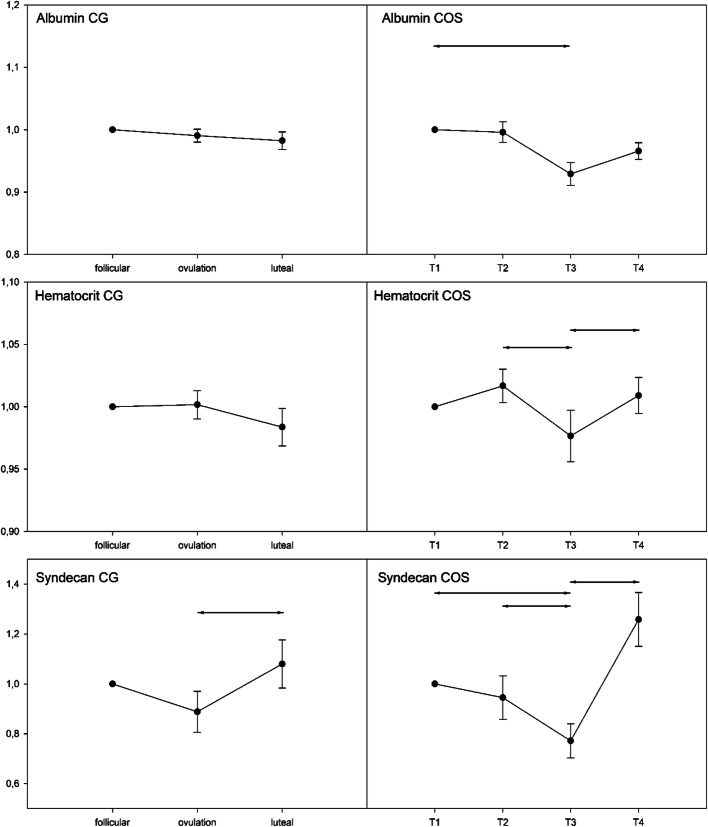
EGX shedding and hemoconcentration. Relative change of albumin, Syndecan-1 serum concentrations, and hematocrit. CG: control group—Syndecan-1 serum levels differed significantly from ovulation to mid-luteal phase (*p=0.020). Albumin serum levels and hematocrit did not change significant over time. COS: controlled ovarian stimulation group—Syndecan-1 serum levels differed significantly from T1 to T3 (p=0.01), from T2 to T3 (p=0.035) and from T3 to T4 (p=0.003). Albumin serum levels differed significantly from T1 to T3 (*p=0.001). Changes form T3/T4 just missed the significance level (p=0.055). Hematocrit serum levels differed significantly from T2 to T3 (*p=0.006) and from T3 to T4 (#p=0.027). Line: The line is representing the mean, the error bars show the standard error of mean. Statistics: ANOVA for repeated measurements

The results concerning albumin and hematocrit levels are depicted in Fig. 2. In the control group, both parameters remained constant. In contrast, we observed significant differences concerning both parameters in group COS. In the estrogen-dominated phase, a downward trend was observed, which led to significant decreases of both values up to T3. The progesterone-dominated phase showed a significant increase of the hematocrit (p = 0.027). Albumin levels also showed an upward trend from T3 to T4 which, in contrast to the hematocrit, just missed the significance level (p = 0.055). The increase in both parameters observed under the influence of progesterone showed a strong correlation (r = 0.43, p < 0.01).

### Spontaneous and inducible hydrogen peroxide release of PMNL

**Fig. 3:**
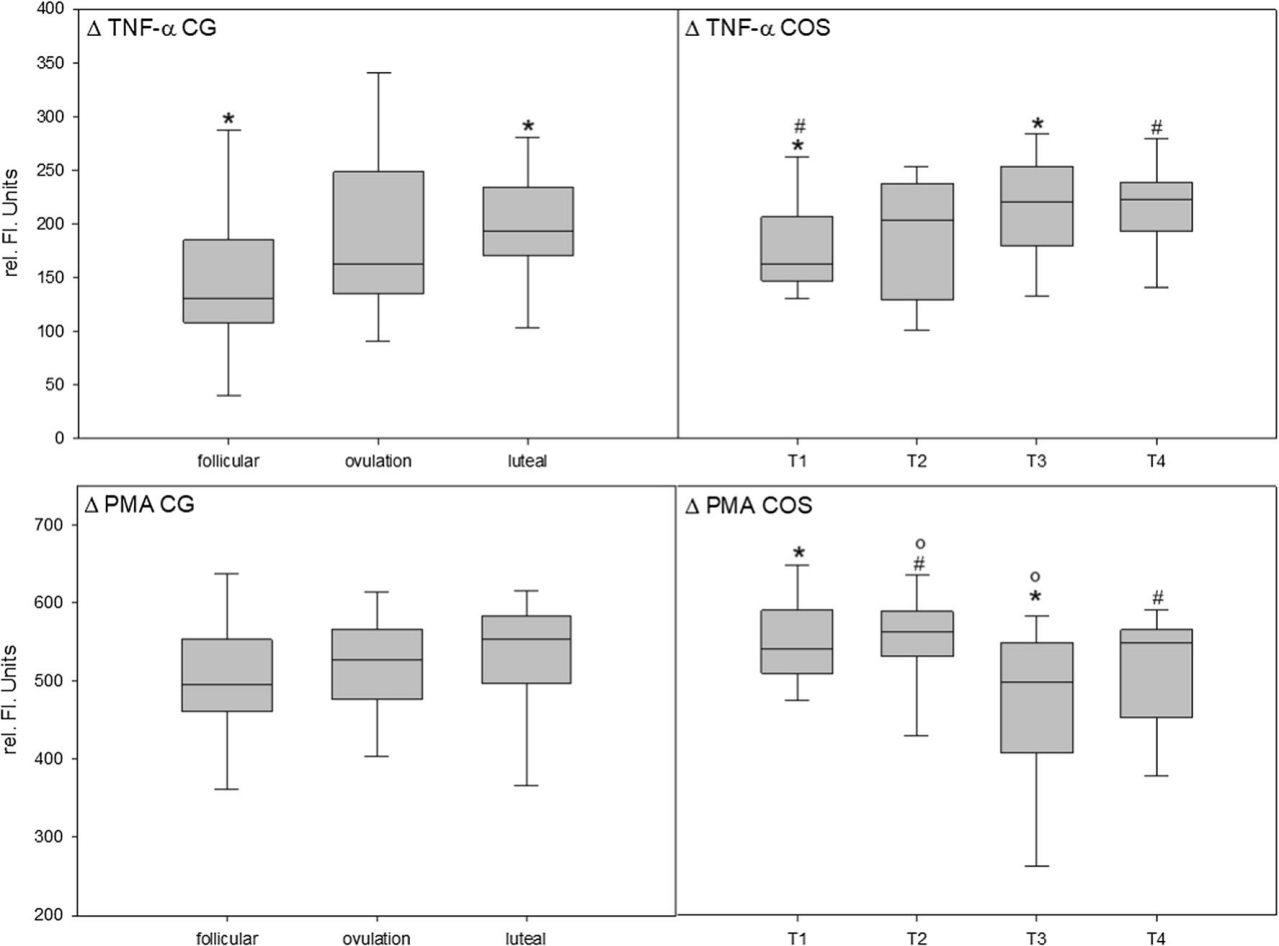
Innate immune system. Polymorphonuclear leukocytes, representing the non-specific immune system, were analyzed for their receptor-dependent (TNF-α) and their receptor-independent (PMA) activity. ΔTNF-α shows the difference between the receptor-dependent and the spontaneous activity. ΔPMA shows the difference between the receptor-independent and the spontaneous activity as expressed in rel. fl. units. CG: control group—ΔTNF-α counts differed significantly from follicular to mid-luteal phase (*p= 0.017). COS: controlled ovarian stimulation group—ΔTNF-α counts differed significantly from T1 to T3 (*p=0.005) and from T1 to T4 (#p=0.017). ΔPMA counts differed significantly from T1 to T3 (*p=0.006), from T2 to T3 (°p=0.007), and from T2 to T3 (#p=0.031). Boxplots: The horizontal line in between the box is representing the median, the whiskers show the 5th/95th percentile. Statistics: ANOVA for repeated measurements

The results concerning the spontaneous activity, the TNF-α-triggered receptor-dependent activity and the PMA-triggered receptor-independent activity of the PMNL, are shown in Fig. 3. Receptor-dependent response to stimulation increased significantly over the observation period in the CG as well as in the COS group.

The intergroup comparison showed a significant difference between groups in the receptor-dependent activity of the PMNL. Starting from comparable values, there were significant differences concerning the measurement times T3 (p = 0.017) and T4 (p = 0.027) compared to the luteal phase of the CG. The receptor-dependent hydrogen peroxide release proved to be about 15% higher in the COS group than in the CG.

## Discussion

In the current study, constantly rising serum levels of estrogen and progesterone were measured during COS, which corresponded to levels shown in early pregnancy [27]. Hofmann-Kiefer et al., who measured EGX components during normal pregnancy, observed constantly rising serum levels of Syndecan-1 and hyaluronic acid over time [28]. Especially in late pregnancy, they found significantly higher serum levels of sex hormones compared to our results [27]. Assuming luteal phase components to be the damaging noxae to the EGX, it was questionable whether comparable EGX component levels could be expected in the current trial.

### Serum concentrations of EGX components

Measured values and variances were comparable to other investigations in terms of serum EGX components [7, 28–30]. Influenced by the luteal phase constituents, the course of Syndecan-1 levels in both groups reproduces the results of our previous CYCLOCALYX I trial and suggests a destructive effect on the EGX [13]. In this study, we were able to demonstrate in a translational approach both the negative influence of progesterone on EGX components measurable in serum and, in an experimental setting, that progesterone leads to the degradation of the EGX. Comparable to the CYCLOCALYX I study, the estrogen-dominated phases appear to have a neutral to protective effect on EGX [13]. Above all, the courses of albumin serum levels and the hemogram support this thesis (Fig. 2). While no significant changes in albumin concentration and hematocrit could be identified in the CG, these parameters showed significant changes over time in the COS. Both parameters significantly decreased prior to oocyte retrieval, parallel to the increase in serum estrogen levels (T2/T3) before finally (T3/T4) re-increasing in line with the rapid increase in progesterone serum concentrations. Relating to the EGX, decreasing EGX component levels were measured in line to decreasing hematocrit and albumin levels, while increasing EGX component concentrations were observed in line to increasing hematocrit and albumin levels. Although albumin has a net negative charge, its amphoteric nature promotes tight binding to the glycocalyx with the net effect of reducing hydraulic conductivity across the vascular barrier, resisting glycocalyx degradation (i.e., protecting against shedding) and thereby contributing to maintenance of vascular integrity and normal capillary permeability [31]. A relevant proportion of plasma albumin is therefore integrated in the structure of the EGX and thus cannot be measured within the normal laboratory routine. Since there is no storage of albumin in the liver, it cannot be just released on demand. However, it is well known that the reservoir of essential plasma proteins embedded in the EGX releases soluble molecules, especially albumin, in the event of EGX degradation [17, 32]. In a clinical setting, the amount of EGX components released as well as the amount of plasma proteins released may provide an indication of the severity of EGX damage [33]. It can be assumed that the rising albumin concentrations in the progesterone-dominated luteal phase, as overserved in the current study, are indeed caused by a degradation of the EGX. The initial changes of the architecture of the EGX at the beginning of its degradation induce different fluid shifts between intravascular and extravascular components. In this context, it is important to know that the albumin molecules, which are embedded in the EGX, are the most important factor in order to keep up the oncotic pressure gradient between the intravascular space and the interstitial space. As a consequence, a decline in the albumin bound in the EGX due to shedding of the EGX should result in a fluid shift out of the intravascular space into the extravascular space. This can be explained by a decreasing oncotic pressure gradient between the EGX and the sub-glycocalyx space, in the form of small endothelial gaps in the junction strand, a “space” between the EGX and the tissue surface. Additionally, however, it has to be considered that the EGX itself fixes a considerable amount of plasma in form of a non-circulating intravascular fluid, the ESL [24]. As a consequence, if there is a degradation of the EGX, the expected intravascular fluid loss into the interstitial space counteracts to a simultaneous mobilization plasma out of the ESL into the circulation. The ratio of these two fluid shifts—on the one hand, extravasation from the vessel as intravascular volume loss and, on the other hand, the release of plasma from the ESL and thus an increase in the intravascular volume—is therefore crucial for the quantitative assessment of the changes in intravascular volume status. Increased extravasation is clinically equivalent to the development of edema, which can be regularly observed within normal ovulatory cycle and COS [34]. Based on these observations, it can be hypothesized that the symptoms of OHSS might be due to a collapse of the ESL, which would explain the symptoms of the OHSS with hemoconcentration, elevated albumin levels, and fluid extravasation.

The non-significant decrease in Syndecan-1 and heparan sulfate levels observed under the influence of follicular phase components contrasts with an increase in hyaluronic acid levels. Granulosa cells as endothelial cells of the ovarian follicle react with an overexpression of hyaluronic acid to the influence of FSH. It is quite conceivable that the values shown reflect this effect, which is more pronounced under the high effect levels of COS [35]. However, further research is needed to clarify this hypothesis.

### Effects of hormone concentrations on the non-specific immune system

Several studies describe the involvement of the EGX in inflammatory processes [16, 30, 36, 37]. Inflammation leads to increased shedding of the EGX compared to normal turnover. The consequence is a relevant increase of EGX components circulating in the blood, a decrease of the EGX’s diameter, a disruption of its integrity, and—consecutively—an impaired functionality. Both the normal ovulatory cycle and the COS are discussed as a sterile inflammatory condition [38–41]. This condition is primarily characterized by increased activity of the non-specific and reduced activity of the specific immune system [42, 43]. During the LH peak and immediately before menstruation, there is a flow of inflammatory cells, predominantly leukocytes into the endometrium [34, 44, 45]. Especially PMNLs are present in the menstrual phase endometrium [46]. Orvieto et al. identified increased activity of the immune system during COS. They found increased levels of markers of inflammation like CRP, leukocytes, and endothelial selectins in blood [38–40]. Of particular interest for the current study was that progesterone and TNF-α serum levels showed a positive correlation while estrogen led to decreasing TNF-α levels [47, 48]. The impact of the pro-inflammatory cytokine TNF-α on the EGX is described as an increased shedding, resulting in the damage of the vascular barrier and an increased extravasation of macromolecules [14, 16]. Although some studies describe the number of leukocytes in the progesterone-dominated luteal phase, only a few studies investigated concurrent changes in the functionality of the PMNL. For three reasons, we expected to find an impact on the PMNL in the COS group. Firstly, Giuliani et al. showed an increased migration number of PMNL during COS, which is accepted as a reliable marker for the effectiveness of the PMNL-based immune system [49]. Secondly, Ficicioglu et al. showed significantly elevated TNF-α serum levels during COS compared to that in a normal menstrual cycle [50]. Thirdly, congruent to our recent findings, we expected higher EGX component concentrations in serum during COS, which in turn can activate leukocytes themselves [51, 52]. Indeed, increased functionality was shown as a function of a significantly increased receptor-dependent functionality of the PMNL over the observation period. But this effect may not with certainty be attributed to estrogen or progesterone alone. With the onset and hence the influence of estrogen, the effect seems to intensify under the influence of the luteal phase constituents, as the intergroup comparison showed a significant difference in this phase.

CG shows no significant difference over the observation period with respect to receptor-independent stimulus response. According to the trend, however, this tends to increase. In contrast, the COS group shows fluctuating to decreasing values over the observation period.

However, the hypothesis that higher sex hormone levels lead to a higher receptor-mediated oxidative burst of PMNL, as well as an influenceability of the receptor-independent stimulus response, is a promising hypothesis that needs to be investigated in further studies.

## Conclusion

Shedding of the EGX under controlled hormonal stimulation with sexual hormones has not yet been described. The current study demonstrates that sexual hormones may play a role in the integrity of the EGX, fluid balance, and functionality of PMNL. Whether this mechanism could be regarded as being the origin behind the clinical consequences of OHSS, including edema, inflammation, and coagulopathy, has to be investigated in further studies.
